# Comparative Assessment Between the Fundus-First Technique and Standard Laparoscopic Technique in Difficult Laparoscopic Cholecystectomy

**DOI:** 10.7759/cureus.74842

**Published:** 2024-11-30

**Authors:** Gaurav Bhoopathy, Monali Priyadarshini, Debendra K Hota, Saroj K Sahoo

**Affiliations:** 1 Department of General Surgery, Kalinga Institute of Medical Sciences, Bhubaneswar, IND

**Keywords:** bailout, cholecystectomy, fundus first, gall stone disease, laparoscopic technique

## Abstract

Background: Laparoscopic cholecystectomy is a standard minimally invasive technique for the treatment in gallstone disease. In difficult laparoscopic cholecystectomies, bailout strategies have been developed of which the fundus-first technique is one. The present study aims to compare the outcomes of the fundus-first technique against the standard laparoscopic approach in managing difficult cholecystectomy cases by focusing on intraoperative factors such as bleeding, bile duct injury, operative time, and postoperative complications like biliary leakage.

Methods: A prospective comparative study was conducted over a period of two years (June 2022-May 2024) with 200 consecutive patients. All patients were classified as difficult cases based on the Tokyo 2018 guidelines. Detailed data collection included patient history, physical examination, laboratory investigations, and operative findings. The study's follow-up period was six weeks.

Results: The fundus-first technique demonstrated significant advantages, with a reduction in operative time, fewer intraoperative complications, and better operative outcomes compared to the standard approach. Patients in the fundus-first group had a mean operative time of 91.50 minutes as compared to 143.75 minutes in the standard group (p < 0.001) and 88% completed operative outcome vs 51%(p < 0.001). No significant differences were observed in postoperative complications such as bile leakage and bleeding between the two groups.

Conclusion: The fundus-first technique offers a safer and more efficient alternative to the standard approach in difficult laparoscopic cholecystectomy cases. By allowing better access to Calot’s triangle, this method proves to be an effective bailout strategy, despite its steep learning curve. Further studies are needed to validate these findings and explore the broader application of the fundus-first technique in gallbladder surgeries.

## Introduction

Cholelithiasis is the most frequent benign disorder of the gallbladder, with a global prevalence of 6% in men and 9% in women. Globally, 6% of the population have gallstones, with higher rates in female gender and South America [1]. The staple surgery used to be open cholecystectomy until the advent of laparoscopic cholecystectomy. Using this technique, gallbladder surgery has become less invasive using only minimal access ports to gain enough working space within the abdominal cavity. The ease of access and the standardization of the technique have made it the standard approach to surgery in cholelithiasis and cholecystitis.

Laparoscopic cholecystectomy has challenges that were not present in open surgery, particularly in difficult cases, which required surgeons to develop alternative approaches known as "bailout methods." One such bailout method is the fundus-first technique. This method, traditionally used in difficult open cholecystectomy cases, involves starting the dissection from the fundus of the gallbladder rather than targeting the neck and Calot’s triangle [2]. Also known as the fundus-down technique, it brings the retracted gallbladder fundus down from the usual position, allowing us to begin dissection at the fundus of the gallbladder, hence the name fundus first. By doing so, surgeons can deal with the challenging Calot’s triangle later in dissection, reducing the need for subtotal resection, preserving the neck's safety margin, and minimizing vasculobiliary injuries. The technique also lowers the likelihood of converting to open surgery. A requirement of a steep learning curve makes it less favored than the easier-to-learn subtotal approach. While the subtotal approach is more straightforward, it does not provide the variety of options that the fundus-first method offers for managing difficult Calot's triangle.

This study aims to complete difficult laparoscopic cholecystectomies using the fundus-first technique and compare the outcomes with the standard laparoscopic approach, focusing on both intraoperative and postoperative complications. Intraoperative factors considered were bleeding, biliary leakage and bile duct injury, operative time, and operative outcomes, while postoperative factors considered were postoperative bleeding and bile leak.

## Materials and methods

Study settings

A prospective comparative study was conducted over a period of two years (June 2022 - May 2024) with 200 consecutive patients admitted to the Department of General Surgery with ethical approval (IEC no. KIIT/KIMS/IEC 1008/2022) for elective cholecystectomy with signed consent.

Population

These patients were divided into two groups: those managed using the fundus-first method (Group 1) and those managed using the standard technique (Group 2).

Inclusion and Exclusion Criteria

Patients included in the study were over the age of 18 diagnosed with cholelithiasis, undergoing elective laparoscopic cholecystectomy and identified as difficult cases based on the Tokyo 2018 guidelines [3].

Patients excluded from the study were those unsuitable for the laparoscopic approach due to coexisting conditions such as bile duct stones, altered liver function tests, suspected gallbladder carcinoma, repeated acute pancreatitis (due to gallstones or alcohol), perihepatic subphrenic adhesions, cirrhosis of the liver, abdominal tuberculosis, previous history of emergency or elective upper abdominal surgery, or altered body habitus such as a narrow costal angle, kyphosis, or scoliosis, with severe morbid obesity (body mass index > 25), severe cardiorespiratory, renal or hepatic decompensation, those with an American Society of Anesthesiologists grade III or higher, uncontrolled coagulopathy or coexistent myeloproliferative and hematologic diseases, and those with gallstones and other benign surgical diseases (e.g., umbilical or inguinal hernia) opting for simultaneous surgery.

Preoperative and operative procedures

Detailed history and physical examination along with routine preoperative investigative workup was done as per hospital protocol (Table 1). If the case was declared difficult by expert surgeons who routinely encounter difficult cholecystectomies on the table, operative findings were noted, and the operating surgeon proceeded with their preferred technique based on their expertise. Operative time, intraoperative bleeding, bile leaks, and bile duct injury rates were recorded.

**Table 1:** Intraoperative findings as per the Tokyo Guidelines 2018

Intraoperative findings	Score
A. Factors related to inflammation of the gallbladder	
Appearance around the gallbladder	
1. Fibrotic adhesions around the gallbladder due to inflammation	2
2. Partial scarring adhesions around the gallbladder	2
3. Diffuse scarring adhesions around the gallbladder	4
Appearance of the Calot’s triangle area	
4. Sparse fibrotic change in the Calot’s triangle area	2
5. Dense fibrotic change but no scarring in the Calot’s triangle area	3
6. Partial scarring in the Calot’s triangle area	4
7. Diffuse scarring in the Calot’s triangle area	5
Appearance of the gallbladder bed	
8. Sparse fibrotic change in the gallbladder bed	1
9. Dense fibrotic change but no scarring in the gallbladder bed	2
10. Partial scarring in the gallbladder bed	3
11. Diffuse scarring in the gallbladder bed (includes atrophic gallbladder with no lumen due to severe contraction)	4
Additional findings of the gallbladder and its surroundings	
12. Edematous change around the gallbladder/in the Calot’s triangle area/in the gallbladder bed	1
13. Easy bleeding at dissection around the gallbladder/in the Calot’s triangle area/in the gallbladder bed	3
14. Necrotic changes around the gallbladder/in the Calot’s triangle area/in the gallbladder bed	4
15. Non-iatrogenic, perforated gallbladder wall and/or abscess formation towards the abdominal cavity noted during adhesiolysis around the gallbladder	3
16. Abscess formation toward the liver parenchyma	4
17. Cholecysto-enteric fistula	5
18. Cholecysto-choledochal fistula (included in the expanded classification of Mirizzi syndrome)	6
19. Impacted gallstone in the confluence of the cystic, common hepatic, and common bile duct (included in the expanded classification of Mirizzi syndrome)	5
B. Intra-abdominal factors unrelated to inflammation	
20. Excessive visceral fat	2
21. Inversion of the gallbladder in the gallbladder bed due to liver cirrhosis	4
22. Collateral vein formation due to liver cirrhosis	4
23. Noninflammatory (physiological) adhesion around the gallbladder	1
24. Anomalous bile duct	4
25. Gallbladder neck mounting on the common bile duct	3”

Postoperative Care and Follow-Up

Postoperative care followed the operating hospital's protocol, and incidents of bleeding and bile leaks in the postoperative period were recorded. A follow-up was done after six weeks to assess the overall clinical well-being of the patient. No leading questions were asked unless the patient brought up specific concerns. 

Group 1: Pre- and postoperative protocols were followed uniformly. This group consisted of cases managed using the fundus-first dissection technique after being declared difficult on the table. The approach started at the fundus of the gallbladder, completing the posterior dissection before approaching the Calot's region. The fundus-first technique allowed posterior access to the neck region, facilitating the completion of dissection of the Calot's triangle and the separation and clipping of the cystic artery and duct. After complete separation, the specimen was placed in an endobag and sent for histopathological examination.

**Figure 1:**
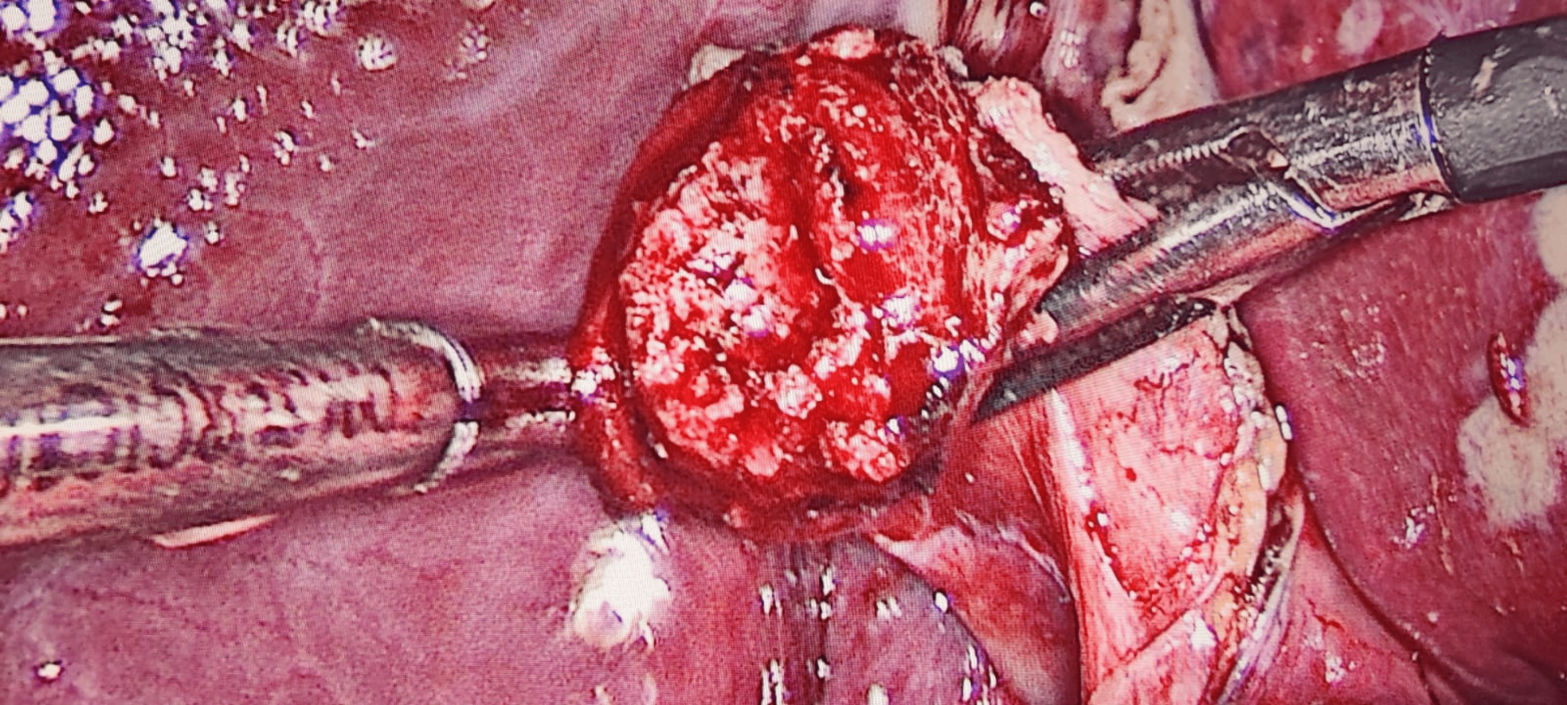
Elective opening of the gallbladder and separation of the neck by submucosal dissection

**Figure 2:**
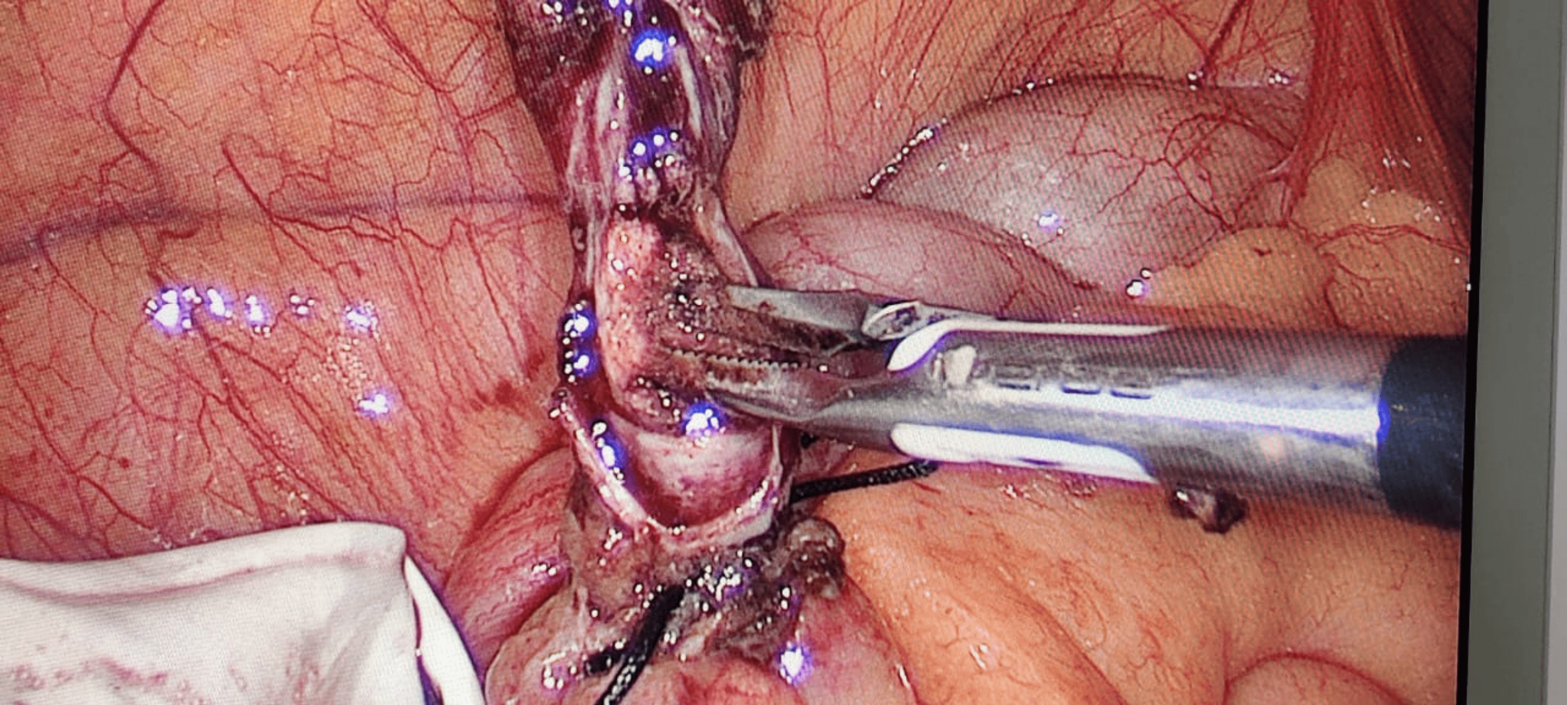
Reconstituted neck being ligated

In cases of densely adherent or chitinous gallbladder, elective partial cholecystectomy was performed leaving the posterior wall in situ if required, with the neck region managed by reconstituting procedure as needed. The reconstitution procedure done was a modified technique from the B type of reconstituting procedure where a submucosal dissection was done (Figure 1) at the neck part to allow for a full reconstitution of the neck, and then the neck was either sutured or with an extracorporeal knot placed (Figure 2)[4].

Group 2: Pre- and postoperative protocols were followed uniformly. This group included cases approached primarily from the Calot's area, with anterior adhesiolysis performed to release the cystic artery and duct region. If successful, posterior dissection was completed, and the specimen was extracted using an endobag. In case of failure a subtotal technique of either leaving the posterior wall in situ with cauterization of the mucosal surface or removing the fundus with the stone complex was performed. The specimen was then placed in an endobag and extracted.

Sample size: Based on the patient recruitment criteria, a total number of 100 consecutive cases were included in Group 1, and for comparison, 100 cases were included in Group 2 in a 1:1 ratio.

Statistical analysis was done using the IBM SPSS Statistics for Windows, Version 21 (Released 2012; IBM Corp., Armonk, New York, United States). The summary of continuous variables has been presented in means ± standard deviation, while the summary of categorical variables is given in frequencies (%).

Student t test was applied for comparative analysis. Chi-square test was used for categorical data. A p-value of <0.05 was applied for the level of significance.

## Results

Preoperative parameters

Demographically, the mean age was 45.02 in Group 1 and 49.46 in Group 2 with 32% of Group 1 being male patients, while Group 2 had 42% of male patients. Clinically, similar histories were noted in terms of pain (70% vs. 69%), abdominal distention (5% each), vomiting (70% vs 69% mild and 30% vs 29% moderate to severe), fever (34% vs 33%), comorbid conditions (21% hypertensive and 20% diabetic in each) and prior surgical histories (13% prior lower segment Caesarean section vs 12% prior lower segment Caesarean section). On clinical examination, all patients were dehydrated in both groups, and pallor was noted in 68% in Group 1 and 66% in Group 2 patients. The mean body mass index was 18.39 in Group 1 while in Group 2 was 19.14. Abdominal distention was present in 5% patients. No gallbladder lump was palpable nor was paralytic ileus present in any of the patients. Biochemical parameters showed a mean hemoglobin of 8.98 in Group 1 and 9.07 in Group 2, while the mean total leukocyte count was 12.57 in both groups. Sugar profiles, liver function tests, coagulation profiles, and renal function tests of both groups were similar. Radiological findings showed thickened, edematous gallbladder in 23% of both groups and contracted gallbladder in 77% of both groups as well. A total of 20% of both groups had fatty liver, while the other ultrasound findings were normal (Table 2).

**Table 2:** Preoperative parameters LSCS: lower segment Caesarean section; BMI: body mass index; Hb: hemoglobin; TLC: total leukocyte count; FBS: fasting blood sugar; PPBS: postprandial blood sugar; RBS: random blood sugar; HbA1c: glycated hemoglobin; Na^+^: sodium; K^+^: potassium; bilirubin (D): direct bilirubin; bilirubin (I): indirect bilirubin; AST (SGOT): aspartate aminotransferase (serum glutamic oxaloacetic transaminase); ALT (SGPT): alanine aminotransferase (serum glutamate pyruvate transaminase); ALP: alkaline phosphatase; GGT: gamma glutamyl transferase; PT: prothrombin time; INR: international normalized ratio; CBD: common bile duct

Data	Fundus-first group	Standard group	p-value
Demographic data			
Age	45.02 ± 15.19	49.46 ± 14.98	0.039
Male	32%	42%	0.187
Female	68%	58%	0.187
Clinical parameters			
Pain: mild	70%	69%	0.999
Pain: moderate to severe	30%	31%	0.999
Abdominal distention	5%	5%	1
Vomiting: mild	70%	69%	0.999
Vomiting: moderate/severe	30%	31%	0.999
Fever	34%	33%	0.999
Hypertensive	21%	21%	0.999
Diabetic	20%	20%	0.999
Prior surgical history (LSCS)	13%	12%	0.828
Dehydration	100%	100%	1
Pallor	68%	66%	0.881
BMI	18.39 ± 1.92	19.14 ± 2.01	0.008
GB lump absent	100%	100%	1
Paralytic ileus absent	100%	100%	1
Biochemical parameters			
Hb	8.98 ± 1.68	9.07 ± 1.67	0.68
TLC	12.57 ± 3.01	12.57 ± 3.01	0.99
FBS	103.29 ± 35.05	103.41 ± 35.02	0.981
PPBS	147.22 ± 42.75	147.15 ± 42.79	0.991
RBS	111.44 ± 56.65	111.42 ± 56.65	0.998
HbA1c	5.15 ± 1.40	5.15 ± 1.40	0.999
Na+	139.48 ± 6.36	139.55 ± 6.44	0.938
K+	4.51 ± 0.50	4.51 ± 0.50	0.888
Urea	12.89 ± 4.67	12.84 ± 4.62	0.939
Creatinine	0.89 ± 0.22	0.90 ± 0.22	0.950
Bilirubin (D)	0.93 ± 0.21	0.93 ± 0.21	0.990
Bilirubin (I)	0.89 ± 0.23	0.90 ± 0.23	0.852
AST (SGOT)	30.79 ± 3.55	30.82 ± 3.55	0.952
ALT (SGPT)	22.56 ± 4.47	22.50 ± 4.44	0.924
ALP	92.61 ± 33.68	93.08 ± 33.61	0.921
GGT	28.05 ± 12.64	27.92 ± 12.67	0.942
Albumin	2.44 ± 1.00	2.44 ± 1.00	0.992
PT	12.27 ± 0.39	12.27 ± 0.39	0.984
INR	1.15 ± 0.08	1.15 ± 0.87	0.935
Radiological data			
Fatty liver	20%	20%	1
Gallbladder contracted	77%	77%	1
Gallbladder thickened and edematous	23%	23%	1
CBD normal	100%	100%	1
Pancreas normal	100%	100%	1
Spleen normal	100%	100%	1

Intraoperative findings and operative outcomes

All cases of Groups 1 and 2 were declared difficult on table. Intraoperatively, findings in Group 1 showed predominantly a score of 4 surrounding the gallbladder (97%) and a score of 2 (3%) secondarily, while in the Calot’s triangle, a score of 4 was also noted primarily (88%) with scores of 3 (9%) and 2 (3%) being less frequent. The gallbladder fossa scores were noted as a score of 3 predominantly (88%) with scores of 4 (9%) and 2 (3%) being less frequent. Additional findings showed a score of 3 in all cases with other intra-abdominal factors being scored at 2 (77%) predominantly and 3 (23%) being less frequent. In Group 2, however, score 4 although predominant (78%) had a significant difference from Group 1, while score 2 (22%) was noted more frequently than in Group 1. The Calot’s triangle was also significantly different with a score of 3 being the foremost (78%) with a score of 2 being more frequent than in Group 1 as well (22%). Gallbladder fossa findings were also significantly different with score 4 being noted mostly (78%) and score 2 (22%) being noted more frequently than in Group 1. Additional findings and other intra-abdominal findings were the same as in Group 1. Intraoperatively bleeding was noted in 23% of Group 1 cases and 22% of Group 2 cases. Bile duct injury was not seen in any of the cases. Minimal bile leak, however, was noted in 22% of Group 2 cases, while Group 1 had no bile leak. A significant difference was noted in the operative time with a mean of 91.50 minutes being taken in Group 1 with 143.75 minutes being taken in Group 2. Operative outcomes were also significantly different with completion being achieved in 88% of cases in Group 1 with partial in 12%, while in Group 2 only 51% completion was achieved with subtotal resection done in 49% of cases (Table 3).

**Table 3:** Intraoperative findings and operative outcomes GB: gallbladder

Data	Fundus-first group	Standard group	p-value
Intraoperative difficulty	100%	100%	1
Surrounding GB: score 2	3%	22%	<0.001
Surrounding GB: score 4	97%	78%	<0.001
Calot's triangle: score 2	3%	22%	<0.001
Calot's triangle: score 3	9%	78%	<0.001
Calot's triangle: score 4	88%	0%	<0.001
GB fossa: score 2	3%	22%	<0.001
GB fossa: score 3	88%	0%	<0.001
GB fossa: score 4	9%	78%	<0.001
Additional finding: score 3	100%	100%	1
Other intra-abdominal factors: score 2	77%	77%	0.999
Other intra-abdominal factors: score 3	23%	23%	0.999
Intraoperative bleeding: mild to moderate	23%	22%	0.999
Bile duct injury	100%	100%	1
Bile leak: minimal	0%	22%	<0.001
Bile leak: absent	100%	78%	<0.001
Operative time	91.50 ± 18.30 min	143.75 ± 13.70 min	<0.001
Operative outcome: complete	;88%	51%	<0.001
Operative outcome: partial	12%	0%	<0.001
Operative outcome: subtotal	0%	49%	<0.001

Postoperative findings

Postoperative bleeding and bile leak was not noted in either group, and both groups had equally adequately healthy postoperative follow-ups (Table 4).

**Table 4:** Postoperative findings

Data	Fundus-first group	Standard group	p-value
Postop bleeding	0%	0%	1
Postop bile leak	0%	0%	1
Follow-up: good	100%	100%	1

## Discussion

A newer procedure was employed in our Group 1 which was done in 12% of cases where a modification of the B type of subtotal procedure was done in that subset of cases with a chitinous posterior wall [4]. In these cases, the repeated or severe inflammation leads to a densely adherent posterior wall which is inseparable from the gallbladder fossa. In such cases, the literature describes a procedure where the posterior wall is left in situ with the evacuation of the stones and contents of the gallbladder while reconstituting a still attached neck [5].

In Group 1, we employed a submucosal dissection technique to separate the neck. This is supported by the remnant vascularity to the neck from short posterior branches arising from the cystic bed. The separated neck now was divided from the body of the gallbladder and was reconstituted with endosuturing or extracorporeal knotting. This method was facilitated by the fundus-down approach as it gives a top-down approach to the neck, which allows hooking of the neck from the posterior side. This approach allowed a reduction in the bile leak from the remnant stump which was not possible in the standard technique.

A study comparing the safety of the fundus-first laparoscopic cholecystectomy to the standard approach using data from the Swedish GallRiks registry by Edergren et al. [6] analyzed over 163,500 surgeries performed from 2006 to 2020. Surgeons were categorized based on their preference for a fundus-first laparoscopic cholecystectomy: less than 20%, 20%-79%, and 80% or more of their cases. The study found no significant differences in the overall incidence of surgical complications or bile duct injuries between the groups. Interestingly, the group with surgeons predominantly performing fundus-first laparoscopic cholecystectomy (≥80% of cases) showed significantly lower rates of bleeding and gallbladder perforation and had shorter operative times. The findings indicate that both the fundus-first laparoscopic cholecystectomy and the standard approach are safe techniques for laparoscopic cholecystectomy, with no major differences in complication rates. This suggests that proficiency in both methods can help surgeons effectively manage different case complexities. The study underscores the importance of observational studies for comparing surgical methods, given the challenges of conducting randomized controlled trials in surgical practice.

Our study found similar findings with a shorter operative time being the hallmark of the fundus-first approach. Our study also found that the fundus-first technique allows for higher rates of completion as compared to the standard dissection.

Conversion from laparoscopic cholecystectomy to open cholecystectomy varies due to factors like surgeon experience and the complexity of the procedure. A study by Nassar et al. evaluated the reasons for conversion, strategies to reduce it, and the impact of subspecialization. Analyzing data from 5738 laparoscopic cholecystectomies over 28 years by a single surgeon revealed a 0.49% conversion rate, with higher morbidity at 33%. Key reasons for conversion included dense adhesions and impacted bile duct stones. Salvage techniques like the fundus-first laparoscopic cholecystectomy and subtotal cholecystectomy significantly reduced conversion rates. Prospectively collected data highlighted that conversion often occurred early in the series, suggesting a learning curve effect. Patients with risk factors such as jaundice, previous acute cholecystitis, and dilated common bile ducts had higher conversion rates. The study emphasized routine intraoperative cholangiography and laparoscopic bile duct exploration to clarify biliary anatomy and manage stones effectively. The Nassar difficulty grading scale was used to standardize reporting and facilitate comparison across different surgeons and cases [7].

The findings underscore that while open cholecystectomy should not be viewed as a failure, it carries higher morbidity and should be a last resort. Subspecialization, high emergency case volumes, and advanced techniques can safely lower conversion rates and associated morbidities. The study's results were compared with that of the national data from the CholeS study [8], highlighting the predictors of conversion and emphasizing the benefits of specialized, experienced surgical practice in managing difficult cholecystectomies.

In our study, however, none of the operating surgeons preferred to go for open conversion and decided to use either subtotal cholecystectomy with reconstituting procedure along with fundus-down dissection or leaving the posterior wall in situ or even by the standard method rather than convert to open surgery.

A study by Strasberg et al. in 2012 [9] investigates the mechanism behind extreme vasculobiliary injuries involving major hepatic arteries and portal veins, primarily through the analysis of clinical records and operative notes from eight patients treated between 1999 and 2010. All patients, who were females with a mean age of 56.8 years, exhibited severe gallbladder inflammation, which necessitated conversion from laparoscopic to open cholecystectomy procedures. The fundus-first cholecystectomy approach led to severe bleeding from major portal veins and hepatic arteries in seven patients. These injuries were complex and involved both veins and arteries, resulting in significant complications such as liver infarction.

The outcomes were severe, with four patients requiring a right hepatectomy, one needing a liver transplant, and four succumbing to their injuries. The study concludes that the fundus-down approach in the presence of severe inflammation significantly increases the risk of extreme vasculobiliary injuries. These findings highlight the importance of heightened awareness and cautious surgical techniques when dealing with inflamed gallbladders to prevent these life-threatening complications [9]. This study has been quoted in the 2018 Tokyo Guidelines as well [3].

However, our study indicates that vasculobiliary injury was absent in all cases and that fundus first is a safe and feasible method of bailout with no incidence of bile leak and biliary injury. We adhered to the “Delphi consensus” on staying close to the inflamed gallbladder wall, and minimal bleeding was encountered in 23% of cases which was primarily from the posterior wall remnant [3].

The pitfalls of the study include a single-center study with a limited sample size where the surgeons were given a choice of procedure based on individual preference and competence, and it became more individual skill based than protocol based and could have led to bias.

## Conclusions

The challenge of the difficult neck is the crux of difficult laparoscopic cholecystectomy requiring surgeons to be well versed in multiple options for bailout. The fundus-first method should be considered a primary bailout strategy in difficult laparoscopic cholecystectomy, as it allows to deal with more difficult situations, reduces operative time, improves operative outcomes, and reduces complications, making it an efficient and safer alternative to the standard approach which is the subtotal method.

Major concerns with the fundus-first technique which led to its falling out of favor initially were the fear of extreme injury in overzealous dissection, and the concerns of distorted anatomy have been addressed in the study. The study recommends regular practice of the technique in challenging scenarios to allow surgeons to pick up the technique to add to their repertoire of dealing with a difficult gallbladder neck and emerging with successful outcomes. The decreased time reduces anesthetic exposure time in those patients with other comorbid conditions requiring quicker surgery and better operative outcomes leading to better postoperative periods in the long term with lesser chances of recurrence. Further studies are needed to validate these findings, examine further long-term outcomes, and explore the broader application of the fundus-first technique in laparoscopic cholecystectomy.
